# Epigenetic modulators of B cell fate identified through coupled phenotype-transcriptome analysis

**DOI:** 10.1038/s41418-022-01037-5

**Published:** 2022-07-13

**Authors:** Isabella Y. Kong, Stephanie Trezise, Amanda Light, Izabela Todorovski, Gisela Mir Arnau, Sreeja Gadipally, David Yoannidis, Kaylene J. Simpson, Xueyi Dong, Lachlan Whitehead, Jessica C. Tempany, Anthony J. Farchione, Amania A. Sheikh, Joanna R. Groom, Kelly L. Rogers, Marco J. Herold, Vanessa L. Bryant, Matthew E. Ritchie, Simon N. Willis, Ricky W. Johnstone, Philip D. Hodgkin, Stephen L. Nutt, Stephin J. Vervoort, Edwin D. Hawkins

**Affiliations:** 1 Walter and Eliza Hall Institute of Medical Research, 1G Royal Parade, Parkville, 3052 VIC Australia; 2 Department of Medical Biology, The University of Melbourne, Parkville, 3010 VIC Australia; 3 Peter MacCallum Cancer Centre, Melbourne, 3000 VIC Australia; 4 Sir Peter MacCallum Department of Oncology, The University of Melbourne, Melbourne, VIC Australia; 5 Victorian Centre for Functional Genomics, Peter MacCallum Cancer Centre, Melbourne, VIC Australia

**Keywords:** Cell death and immune response, Epigenetics

## Abstract

High-throughput methodologies are the cornerstone of screening approaches to identify novel compounds that regulate immune cell function. To identify novel targeted therapeutics to treat immune disorders and haematological malignancies, there is a need to integrate functional cellular information with the molecular mechanisms that regulate changes in immune cell phenotype. We facilitate this goal by combining quantitative methods for dissecting complex simultaneous cell phenotypic effects with genomic analysis. This combination strategy we term Multiplexed Analysis of Cells sequencing (MAC-seq), a modified version of Digital RNA with perturbation of Genes (DRUGseq). We applied MAC-seq to screen compounds that target the epigenetic machinery of B cells and assess altered humoral immunity by measuring changes in proliferation, survival, differentiation and transcription. This approach revealed that polycomb repressive complex 2 (PRC2) inhibitors promote antibody secreting cell (ASC) differentiation in both murine and human B cells in vitro. This is further validated using T cell-dependent immunization in mice. Functional dissection of downstream effectors of PRC2 using arrayed CRISPR screening uncovered novel regulators of B cell differentiation, including *Mybl1*, *Myof*, *Gas7* and *Atoh8*. Together, our findings demonstrate that integrated phenotype-transcriptome analyses can be effectively combined with drug screening approaches to uncover the molecular circuitry that drives lymphocyte fate decisions.

## Introduction

The production of protective antibodies in response to challenge with pathogens is an essential element of effective immunity. Antibodies are produced by plasma cells (or antibody secreting cells – ASC) that develop from stimulated B cells [1]. ASC differentiation is highly orchestrated, and the molecular steps are well-known [1]. However, the healthy path to ASC formation can be disrupted, resulting in diseases such as immunodeficiency, autoimmunity or the development of blood cancers. Thus, therapeutic strategies that can modulate both ASC differentiation and antibody production are the focus of drug screening approaches.

In recent years, it has become apparent that changes in the epigenetic landscape are associated with the development of both immune disorders and cancer [2]. In turn, epigenetic modifying compounds (EMCs) have emerged as promising therapeutic agents for treating haematological malignancies and immune disorders [3]. The B cell lineage is particularly susceptible to epigenetic modifications through EMCs and has led to clinical trials using compounds such as panobinostat to treat the plasma cell malignancy, myeloma [4]. Despite extensive studies investigating EMCs, the precise functional effects of most of these compounds remain unclear.

Here, we used Multiplexed Analysis of Cells sequencing (MAC-seq) in conjunction with a quantitative framework for dissecting and isolating immune cell function in vitro to simultaneously define the transcriptional and phenotypic effects of selected EMCs. Through this approach, we characterized the effects of 60 EMCs, defining the epigenome mediated influence on ASC differentiation and highlighting a functional and potentially therapeutically targetable role of polycomb repressive complex 2 (PRC2) inhibition in regulating the immune response. Furthermore, the extension of these findings to arrayed CRISPR screens identified novel target genes of PRC2 that regulate ASC differentiation.

## Results

### MAC-seq dissects the effects of epigenetic inhibitors on B cell proliferation, survival and ASC differentiation

**Fig. 1:**
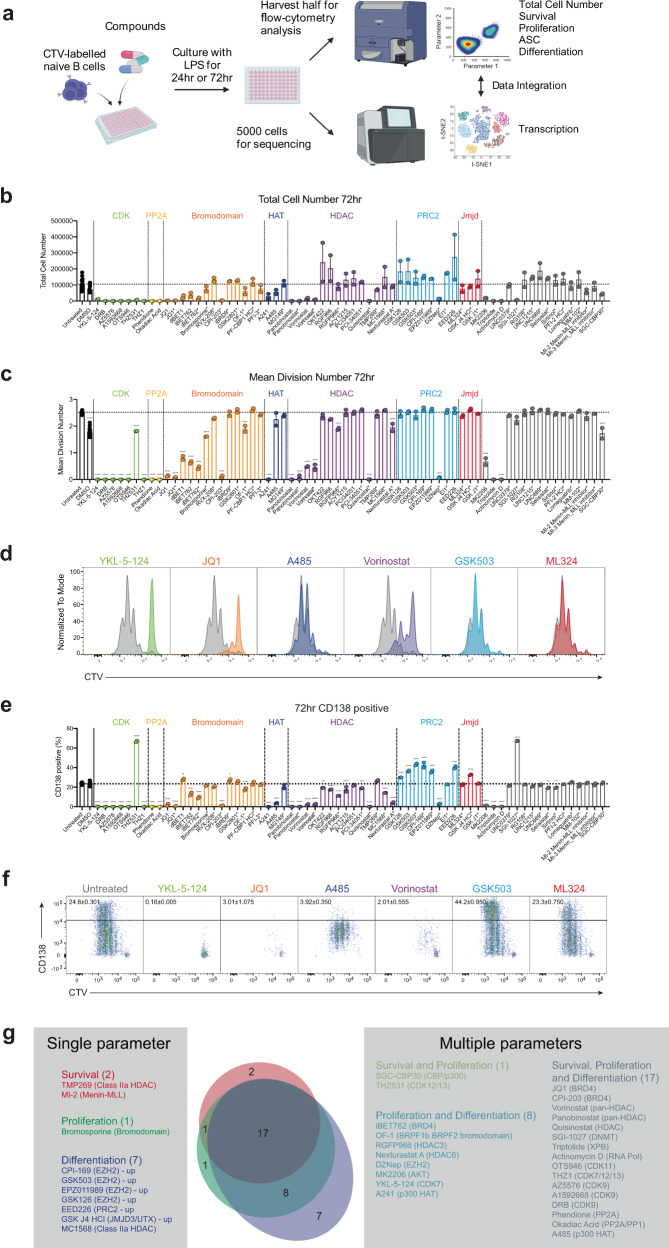
Flow cytometry analysis identifies B cell parameters affected by epigenetic modifying compounds. **a** Experimental workflow. CTV-labeled naïve murine B cells were cultured in LPS and treated with various compounds(1 µM). Half of the culture was harvested for flow cytometry analysis and transcriptome analysis was performed on 5000 cells per condition at 24 hr and 72 hr. **b** Total cell number and **c** mean division number at 72 hr post culture. **d** Representative CTV profiles for YKL-5-124, JQ1, A485, Vorinostat, GSK503 and ML324. Untreated controls are in gray. **e** Percentage of CD138^+^ cells at 72 hr. **f** Representative plots of CD138 expression for untreated, YKL-5-124, JQ1, A485, Vorinostat, GSK503 and ML324. **g** Summary on the effect of compounds on parameters measured by flow cytometry. Data in **d**, **f** are representative of two independent replicates. Significant differences in **b**, **c**, **e** were determined using ANOVA Bonferroni corrections. *p ≤ 0.05, **p ≤ 0.01, ***p ≤ 0.001, ****p ≤ 0.0001.

We performed quantitative functional and epigenetic analysis on a panel of 60 EMCs, including compounds targeting bromodomains, histone acetyltransferases (HATs), histone deacetylases (HDACs) and polycomb repressive complex 2 (PRC2) (Supplementary Table 1). In addition to the 40 EMCs from Compound Australia’s epigenetics library, we included (1)EMCs that have been previously studied functionally in B cells as positive controls [5] and (2)identical compounds from different commercial sources as an internal control that both functional and transcriptional readouts are repeatable. In recent years it has become apparent that several phosphatases play critical roles in the regulation of transcription. PP2A may directly regulate RNA Polymerase II as well as epigenetic components such as BRD4, and it is for this reason that PP2A inhibitors were included in our study [6–8]. For this screen, we cultured murine B cells with 1 µM of the compounds tested, which is the reported concentration that works for many EMCs. To quantify how the B cell response was altered, we combined quantitative flow cytometry to measure changes in proliferation, survival and ASC differentiation with MAC-seq to examine the overall transcriptomic changes (Fig. 1a).

B lymphocytes stimulated by LPS in vitro follow kinetic principles broadly consistent with the general Cyton model [9–13]. Thus, they regulate survival and division independently, and cells display a broad variation in time to first division that accounts for the highly asynchronous division peaks. Furthermore, stimulated cells progressively differentiate to ASC at a frequency that increases with each generation [11, 14]. These features are consistent with the quantitative framework of lymphocyte responses outlined in Fig. S1a [9, 12, 15, 16] and are applied here to dissect and isolate the effects of inhibitor compounds. By reference to these methods, we initially determined that we could extract key features of drug-targeting from a typical 4-day in vitro assay by focussing on two critical timepoints – a 24 h (hr) timepoint to characterize changes restricted to lymphocyte survival and a 72 hr timepoint for B cell activation, proliferation and differentiation (Fig. S1b). Flow cytometry data identified EMCs that significantly reduced total cell numbers at 24 hr before lymphocytes enter division, thus classifying these compounds as modifiers of cell survival **(**Fig. S1c). This effect also manifests as a reduction in total cell number at 72 hr **(**Fig. 1b). By integrating data from both time points, we can infer compounds where the effects on B cell function are restricted solely to lymphocyte survival or broader changes in the immune response. Precursor cohort analysis (that eliminates effects of division on cell numbers) can be integrated into the analysis to determine if reduced cell numbers at 72 hr are only due to altered survival or a result of concurrent changes in proliferation [9] (Fig. S1d). By calculating the mean division number of responding cells over time, proliferation kinetics can be accurately measured. Using this approach, we identified compounds that phenotypically modify cell death, including CDK inhibitors, some bromodomain (JQ1 and CPI-203) and HDAC inhibitors (panobinostat, vorinostat and Quisinostat) (Fig. S1c, d). We also identified compounds that reduced proliferation and ASC differentiation, including CDK, Bromodomain and HDAC inhibitors (Fig. 1c–f). Interestingly, nine compounds, THZ531, GSK-J4-HCl, SGI-1027 and six out of the eight compounds that target the PRC2 complex, significantly increased ASC differentiation (Fig. 1e, f). Importantly, quantitative outputs obtained from large-scale MAC-seq assays using only two timepoints were comparable to data sets with four timepoints (Fig. S1e–g). Thus, reducing the number of time points for MAC-seq does not affect the sensitivity of the assay.

This approach was used to dissect and assign the effects of each compound into separate categories based on their ability to target single or multiple parameters of the lymphocyte responses (Fig. 1g). We identified EMCs that only affect one component of the B cell response, such as PRC2 inhibitors (GSK503, GSK126 and EED226) that boosted ASC differentiation and bromosporine that only reduced B cell proliferation. In contrast, compounds including JQ1 and the pan-HDAC inhibitors vorinostat and panobinostat affected multiple parameters simultaneously (survival, proliferation and ASC differentiation).

### MAC-seq reveals the transcriptome changes that match the phenotype identified by flow-cytometry

**Fig. 2:**
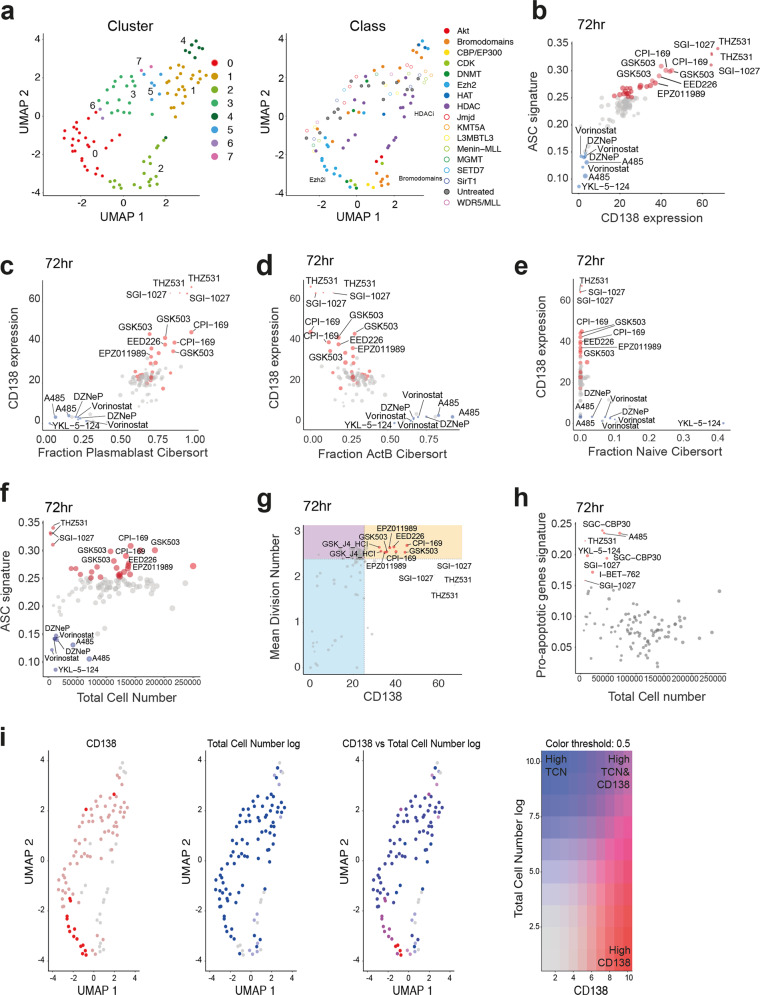
MAC-seq analysis determines transcriptome changes due to epigenetic modifying compounds. CTV-labeled naïve murine B cells were cultured in LPS and treated with various compounds(1 µM). Transcriptome analysis was performed on 5000 cells per condition at 24 hr and 72 hr. **a** UMAP plot for transcriptome data at 72 hr. **b** Expression of CD138 and ASC signature genes at 72 hr. Correlation between CD138 and fraction of (**c**) plasmablasts, (**d**) activated B cells and **e** naïve B cells at 72 hr determined using Cibersortx. **f** Total cell number and ASC signature at 72 hr. **g** CD138 expression and mean division number from Fig. 1 at 72 hr. **h** Total cell number and pro-apoptotic gene signature at 72 hr. **i** UMAP plots highlighting CD138 expression, total cell number (log) and CD138 and total cell number (log) overlapped. Each dot on the UMAP plot represents a compound. Red dots in (**i**) indicate high expression of CD138. Blue dots in **i** indicate high total cell number. Purple dots in **i** indicate high expression of both CD138 and total cell number. ASC signature in **b**, **f** was obtained from GSE60927.

We integrated MAC-seq, a modified version of DRUG-seq [17] to pair quantitative phenotypic changes outlined above with molecular mechanisms regulated by the EMCs. Uniform Manifold Approximation and Projection for Dimension Reduction (UMAP) analysis shows that compounds that target similar classes of proteins cluster closely together (Fig. 2a). We validated the use of CD138 expression to identify compounds that increased ASC differentiation by examining the expression of ASC signature genes [18]. As expected, CD138 expression strongly correlated with the expression of ASC signature genes and was identified as plasmablasts using Cibersortx (Fig. 2b–e, S1h). Consistent with phenotypic parameters, this ASC signature was not observed in 24 hr samples (Fig. S1i). Together, these data show that changes in the transcriptome mirror the phenotypes measured by flow cytometry. This highlights the potential of using transcriptome data to predict changes in the cellular phenotype and the gene-expression networks that underpin these processes in a high-throughput manner.

Although MAC-seq identified compounds that increased ASC differentiation, incorporating a quantitative framework of expected immune cell behaviour was critical for defining compounds with genuine applicability for boosting antibody responses. For example, THZ531 and SGI-1027 significantly increased ASC differentiation (Fig. 2c). However, analysis by reference to the Cyton model demonstrated that survival and proliferation were concurrently impaired in THZ531 and SGI-1027 (Fig. 2f, g, i). We analysed pro-apoptotic gene signatures (Supplementary Table 3) to identify EMCs that induced cell death (Fig. 2h). As expected, EMCs with reduced cell numbers correlated with an increased pro-apoptotic gene signature. Using UMAP plots, we identified compounds with an elevated ASC transcriptional signature, CD138 expression, pro-apoptotic gene signature and total cell number (Fig. S2k). We dissected the data further by examining multiple parameters, including CD138 expression and total cell number to identify compounds that significantly increase ASC differentiation without affecting total cell number (indicated by the purple dots in Fig. 2i). This includes PRC2 inhibitors such as GSK126, GSK503, EED226. Using this approach, we also identified compounds that upregulate both an ASC and pro-apoptotic gene signature, including the compounds THZ531 and SGI-1027 (Fig. S2j).

### Ezh2 inhibition boosts the differentiation of B cells in vitro

**Fig. 3:**
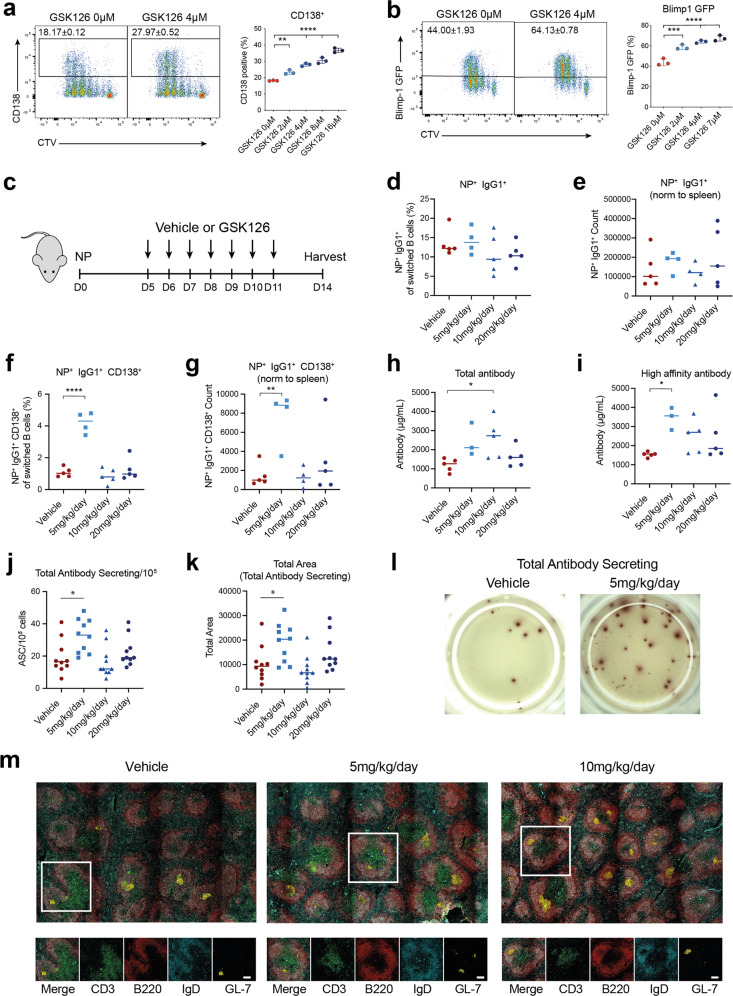
In vitro and in vivo validation for flow cytometry data for Ezh2 inhibitors. **a** CD138 expression and **b** Blimp1-GFP expression murine B cells 72 hr post-stimulation with LPS and indicated concentrations of GSK126. **c** C57BL/6 mice were immunized with NPKLH-Alum and treated with doses of GSK126 as indicated. Spleen and serum samples were analyzed 14 days after immunization. Proportion and total cell number normalized to the spleen for **d**, **e** antigen-specific IgG1 and **f**, **g** antigen-specific ASC. **h** Total antibody and **i** high-affinity antibody measured. **j** Total antibody secreting cells and **k** area of spots detected on ELISpot quantified. **l** Representative wells of ELISpot. **m** Representative images of GC. CD3: T cell zone B220 and IgD: B cell zone; GL-7: germinal center; Vehicle, *n* = 5, 5 mg/kg/day GSK126 treated group, *n* = 4, 10 mg/kg/day and 20 mg/kg/day GSK126 treated groups, *n* = 5. Bars in **m** represents 100 µm. Data in **a**, **b** are representative plots from triplicate samples. Error bars mean±s.e.m. Blinded analysis on germinal centre was performed. All data are representative of three independent experiments. Significant differences were determined using ANOVA with Bonferroni corrections. *p ≤ 0.05, **p ≤ 0.01, ***p ≤ 0.001, ****p ≤ 0.0001.

The observation that PRC2 inhibitors resulted in the potentiation of ASC differentiation without affecting total cell number indicates that these compounds may be used to boost antibody secretion. The effects of PRC2 inhibition on ASC differentiation were examined in greater detail to validate our compound screen. Quantitative analysis was performed on PRC2 inhibitors (GSK126, GSK503 and EED226) in B cell cultures. GSK126 and GSK503 are both potent inhibitors of Ezh2 (the enzymatic catalytic subunit of PRC2) with similar functional properties and very minor structural differences. In contrast EED226 inhibits the H3K27me3 binding pocket of EED, a core component of PRC2. We showed that all PRC2 inhibitors increased ASC differentiation (Fig. 3a, S2a–c). Thus, we performed the detailed validation studies on GSK126 only. As Blimp-1 is readily accepted as a master regulator of ASC differentiation, we examined the effect of GSK126 on upregulation of this transcription factor using transgenic mice that express GFP under the control of the Blimp-1 locus. GSK126 treatment increased the expression of Blimp-1 confirming that GSK126 increases ASC differentiation via the canonical transcriptional pathway (Fig. 3b). This further confirms that GSK126 increases ASC differentiation. To examine whether the effect on ASC differentiation was mediated by Ezh2, the catalytic domain of PRC2, we generated Ezh2 floxed mice that express Cre recombinase under the control of the Fcer2a promoter (CD23^cre^) expressed mature B cells. Thus, Ezh2 was specifically deleted in mature B cells. We observed that B cells isolated from Ezh2^fl/+^CD23^cre^ and Ezh2^fl/fl^CD23^cre^ have an increased CD138 expression in comparison to their wildtype counterparts, confirming increased ASC differentiation is Ezh2 dependent (Fig. S2d, e). GSK126-mediated differentiation was abrogated in the absence of Ezh2, demonstrating that GSK126-mediated ASC differentiation is Ezh2 dependent (Fig. S2f).

We extended our studies to examine if Ezh2 inhibition also increases ASC differentiation in the context of human B cells. As human B cells do not express the main LPS sensing receptor TLR4, purified B cells were stimulated with CD40L + IL21 in the presence GSK126 (an in vitro system that mimics T cell help). Consistent with murine experiments, GSK126 boosted plasma cell differentiation was measured by accumulation of CD27^hi^ CD38^+ve^ ASC (Fig. S2g, h). Division linked differentiation of human B cells to CD27^hi^, CD27^hi^ CD38^+ve^ and CD38^+ve^ ASC (Fig. S2i–l) in response to GSK126 treatment led to a significant increase in detectable total and isotype switched Ig in culture supernatant (Fig. S2m).

### GSK126 treatment increases antigen-specific antibody responses in vivo

We investigated whether inhibiting Ezh2 could be applied therapeutically to boost antibody production in vivo in the context of immunization. We administered GSK126 (either 5, 10 or 20 mg/kg) to mice and measured the antibody response following immunization with the antigen NP-KLH (4-Hydroxy-3-nitrophenylacetyl hapten conjugated to Keyhole Limpet Hemocyanin) [19–21]. As Ezh2 plays an essential role in regulating germinal centre (GC) formation [22], we initiated GSK126 treatment five days post-immunization to ensure efficient GC formation and priming of the immune response and measured the overall effects on day 14 (Fig. 3c) [19]. GSK126 treatment increased the frequency and number of NP-specific ASCs and total serum antibodies, consistent with in vitro findings (Fig. 3f–i). Interestingly, only low doses of GSK126 (5 mg/kg) increases the number of antigen ASCs (Fig. 3f, g). Serum antibody level is also only increased in low doses of GSK126 (5 mg/kg and 10 mg/kg) (Fig. 3h, i). ELISpot suggests that the secretory capacity per cell is also increased for low doses of GSK126 (5 mg/kg) (Fig. 3j–l). This may be due to the toxicity of GSK126 treatment or off-target effects of the compound. We confirmed this phenotype by measuring the effect of higher GSK126 doses on B cell viability. These assays demonstrated that high concentrations of GSK126 causes a reduction in total cell number over time. A reduction in total cell number was observed as early as 24 hr post stimulation, prior to cell division, indicating that the reduction in total cell number is due to cell death (Fig. S2p, q). GSK126 treatment did not affect the frequency and number of NP-specific B cells and memory cells, indicating that Ezh2 inhibition selectively boosts the ASC response (Fig. 3d, e, S2n, o). We performed immunofluorescence analysis of spleen sections to examine effects of GSK126 treatment on the structure of the GC. We showed that the T cell zone (CD3), B cell zone (B220 and IgD) and germinal center (GL-7) are still present when mice were treated with GSK126 (Fig. 3m). The size of GC was also unaffected (Fig. S2r). Thus, our results suggest that inhibiting Ezh2 has the potential as a therapeutic approach to boost antibody responses in vivo.

### GSK126 treatment induces a unique Ezh2-mediated ASC differentiation signature

**Fig. 4:**
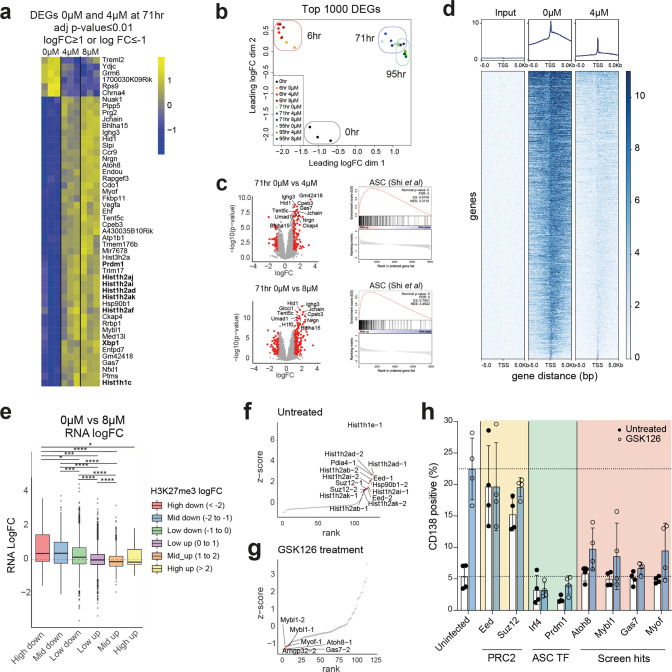
RNA-seq and ChIP-seq to validate MAC-seq data for Ezh2 inhibitors and identifying gene targets of Ezh2 inhibitors. 3’ RNA-Seq was performed on LPS stimulated murine B cells at indicated time points and concentrations of GSK126. **a** Gene expression of DEGs for 0µMvs4µM GSK126 at 71 hr with cut-off values of adj *p*-value ≤ 0.01 and logFC ≥ 1 or ≤ −1. **b** MDS plot for indicated time points and concentrations of GSK126. **c** Volcano plots showing DEGs for 0 µM vs 4 µM and 0µ M vs 8 µM at 71 hr. GSEA analysis of 0 µM vs 4 µM and 0 µM vs 8 µM at 71 hr compared to ASC gene signature (GSE60927). ChIP-sequencing was performed to examine the H3K27me3 occupancy upon treatment with GSK126. **d** H3K27me3 occupancy at TSS regions at 72 hr for untreated and 4 µM GSK126 treated samples. **e** Changes in RNA expression for all genes upon 8 µM GSK126 treatment. Groups are separated based on changes in H3K27me3 upon GSK126 treatment (8 µM). High down indicates logFC < −2. Mid down indicates logFC ≥ −2 and < −1. Low down indicates logFC ≥ −1 and <0. Low up indicates logFC>0 ≤ 1. Mid up indicates logFC>1 and ≤2. High up indicates logFC > 2. **f** Genes that result in an increase in ASC differentiation upon deletion. **g** Genes that abolishes GSK126-mediated ASC differentiation upon deletion. **h** CD138 expression for B cells deleted for the indicated genes for untreated and 4 µM GSK126 treated. Significant differences in **e** were determined using Wilcoxon test. *p ≤ 0.05, ***p ≤ 0.001, ****p ≤ 0.0001.

To validate the transcriptome changes observed in Fig. 1, we performed 3′ RNA-sequencing on B cells treated with GSK126 over time following activation with LPS (0 hr, 6 hr, 71 hr and 95 hr post activation). Significant changes in the transcriptome were dependent on time post-activation with minor changes observed according to treatment with GSK126 (4 µM: 96 DEGs, 8 µM: 245 DEGs; FC ≥ 1 or ≤ −1, adj *p*-value ≤ 0.05) (Fig. 4a, b). We noted significant upregulation of typical ASC differentiation genes such as *Prdm1* (Blimp-1) and *Xbp1* (X-box binding protein 1), consistent with data from Fig. 1. Gene Set Enrichment Analysis (GSEA) also showed a positive enrichment for ASC signatures (GSE60927 and MSigDB M4552) (Fig. 4c and S3a). By comparing GSK126-induced gene signatures to previously characterized ASC genes, we identified several novel Ezh2-regulated genes not previously associated with ASC differentiation, including *Nuak1*, *Atoh8*, *Cdo1*, *Mybl1* and several histone genes (*Hist1h2aj, Hist1h2ai, Hist1h2ad*).

Ezh2 is a critical regulator of epigenetic gene silencing via H3K27-trimethylation. Thus, we investigated whether GSK126-induced changes on the transcriptome are a direct consequence of the inhibition of Ezh2 via H3K27me3. To examine the impact of GSK126 treatment on H3K27me3, we performed chromatin immunoprecipitation (ChIP) sequencing analysis. As expected, GSK126 treatment induced a global downregulation in H3K27me3 levels at both the promoter and enhancer regions (Fig. 4d & S3b–h).

We correlated promoter-associated changes in H3K27me3 with gene expression changes as determined by 3’RNA-seq. We grouped genes based on the magnitude of the H3K27me3 changes in their promoter regions and determined the associated fold-change in RNA expression, revealing an inverse correlation between H3K27me3 and gene expression (Fig. 4e & S3h). These data indicate that PRC2 inhibitor mediated potentiation of ASC differentiation can be attributed to on-target effects and most likely results through modulated H3K27 methylation dynamics at key loci, which drives downstream gene-expression changes.

### Deletion of *Mybl1*, *Myof*, *Gas7* and *Atoh8* abrogates the effect of GSK126 on ASC differentiation

To determine the mechanism of GSK126-mediated ASC differentiation, we performed a functional validation of GSK126 target genes. As Ezh2 inhibition by GSK126 results in the loss of H3K27me3, a mark for gene repression, we hypothesise that the gene targets of GSK126 will be upregulated in response to the treatment. Thus, we performed an arrayed CRISPR screen on the 78 upregulated DEGs in response to 4 µM GSK126 (Fig. S3I, j). To improve the signal-to-noise ratio for the CRISPR screen, we stimulated B cells with LPS + IL-4 that significantly reduces ASC differentiation, increases correlation of the differentiation marker CD138 with bona fide Blimp-1^+^ ASCs [14, 23], and has no effect on GSK126 phenotypes described above (Fig. S3k, l). Following this process, we quantified the effect of targeted genes on ASC differentiation as determined by CD138 expression.

The functional response of all 78 DEGs was tested, and responses were ranked according to differentiation in untreated cultures (Fig. 4f). As expected, deletion of PRC2 components, *Eed* and *Suz12*, increased ASC differentiation and conversely, differentiation was reduced upon deletion of the classically defined regulators of this process, *Irf4* and *Prdm1*(Fig. 4f, h). To identify the critical genetic dependencies of the GSK126-induced ASC differentiation response, we calculated a ranked metric based on the difference in CD138 proportion in response to GSK126 treatment (Fig. 4g). This approach revealed that GSK126-induced ASC differentiation is abrogated upon the deletion of *Mybl1*, *Atoh8*, *Gas7* and *Myof*, indicating that the expression of these genes is required for GSK126-induced ASC differentiation. In addition, the expression of *Atoh8* and *Gas7* in plasma cell subsets has been observed previously (Fig. S4a) [18]. These data suggest that during normal B cell responses, PRC2 may act to suppress these genes, thus limiting the magnitude of ASC differentiation (Fig. 4g, h) [18].

To dissect whether these genes are direct or indirect targets of Ezh2, we examined the status of H3K27me3 of these genes. At basal state, we observed low levels of H3K27me3 at *Gas7*, *Mybl1* and *Myof* and therefore little change in methylation levels upon GSK126 treatment (Fig. S4b, c). *Atoh8* expression is repressed by H3K27me3 in all stages of B cells – activated B cells, pre-plasmablasts and plasmablasts. In plasmablasts, we also noted significant binding of key ASC transcriptional components such as Blimp-1, Irf4 and PU.1 to *Atoh8* (Fig. S4d) [24]. In addition to H3K27me3, the *Atoh8* region is also bound by factors associated with active transcription, including H3K9ac, H3K4me2 and H3K4me3, indicating that the gene is maintained in a poised state (Fig. S4d). Together, these data suggest that the removal of H3K27me3 upon GSK126 treatment releases the repression mark, allowing active transcription of *Atoh8*. These findings correlate with mass spectrometry evidence that Ezh2 is directly bound to Blimp-1, which binds to the *Atoh8* region [24]. To investigate the relationship between Atoh8 and Blimp-1, we performed non-biased statistical methods to examine the expression pattern of these novel genes upon B cell activation in untreated samples and the H3K27me profile of the genes upon B cell activation (Fig. S4e, f). We observed that the expression pattern of *Atoh8* correlates with *Prdm1*, which encodes for Blimp-1 (clusters 6 and 5 respectively – Fig. S4e, g, h, Supplementary Table 2). In addition, the H3K27me3 pattern of *Atoh8* and *Prdm1* fell in the same cluster (cluster 1) with a steady decrease over time (Fig. S4f, i, Supplementary Table 2), suggesting a bona fide relationship between genes associated with ASC differentiation and novel genes such as *Atoh8* identified in this study. Additionally, a recent study by George et al showed that Atoh8  acts as a regulator of M cell differentiation by inhibiting Spi-B [25]. Spi-B is a negative regulator of ASC differentiation and ASC differentiation is increased in mice deficient of Spi-B [26, 27]. Our RNA-seq data also revealed that *Spib* is downregulated in response to GSK126 treatment (Fig. S4j), further suggesting that GSK126 increases ASC differentiation by increasing *Atoh8* expression, which suppresses SpiB.

## Discussion

Using a multi-parameter functional screen of compounds that target epigenetic machinery, we successfully identified drugs that target PRC2 as compounds that boost antibody production. Using this rationale, we functionally validated that these compounds conform to the predictions of our screen both in vitro and in vivo and can be replicated in vitro using human B cells isolated from healthy human donors [28, 29]. Our data revealed that Ezh2 inhibitors increase ASC differentiation, but high concentration of Ezh2 inhibitors induce cell death. We also identified *Mybl1*, *Atoh8*, *Gas7* and *Myof*, as novel regulators of Ezh2-mediated differentiation. While *Mybl1* (that encodes a-Myb), has been linked to the regulation of B cell survival and ASC differentiation [30, 31], *Atoh8, Gas7* and *Myof* have not been reported to be involved in B cell function.

Our results are consistent with the described role of Ezh2 in the regulation of germinal centre formation and lymphomagenesis [22, 32]. Thus, Ezh2 inhibitors are being trialled for efficacy against multiple cancer types with a particular focus on B cell lineage malignancies such as B cell lymphomas [33–35]. Several studies have demonstrated that genes involved in ASC differentiation (including PRDM1) are downregulated in DLBCL subtypes such as activated B cell-like (ABC) DLBCL. PRDM1 is also downregulated in patients with Waldenstrom macroglobulinaemia, which is linked to elevated expression of the negative regulator SPIB [36]. Our results revealed that Ezh2 inhibition increases PRDM1 and downregulates SPIB, suggesting that further investigation of Ezh2 inhibitors in the context of ABC DLBCL and Waldenstrom macroglobulinaemia could be informative. Our findings suggest that PRC2-mediated suppression under homeostatic and inflammatory conditions limits ASC differentiation and that this can be therapeutically exploited through inhibition of Ezh2 to amplify protective antibody responses in various human immune-deficient disorders.

Quantitative frameworks have proven invaluable for the detailed characterization of normal and malignant immune cell populations, providing insights into B and T cell biology [13, 16] and novel approaches to treat T cell malignancies [19]. Independently, high-throughput transcriptional analyses methods, such as MAC-seq, have enabled the dissection of gene-expression networks controlled by genetic or pharmacological targeting. However, a connection between transcriptome and phenotype is imperative to understand how perturbation of immune-cell gene-expression networks give rise to cellular phenotypes. To address this issue, we performed integrated phenotype-transcriptomic analyses by applying quantitative phenotypic frameworks with high-throughput transcriptional characterization using MAC-seq. Our modified version of the protocol highlights the power of this approach as it facilitated novel insights into fundamental mechanisms controlling immune cell biology, delineated the gene-to-phenotype association and simultaneously explored the therapeutic modulation hereof. This study focussed on identifying EMCs that affect B cell responses, particularly the compounds that increase ASC differentiation without affecting the survival and proliferation of lymphocytes. We postulate that this approach will aid in identifying compounds that can be used to modulate specific aspects of the immune response. For instance, boosting ASC differentiation could be beneficial in the context of diseases that manifest as reduced antibody levels (such as immunodeficiency) or dampening the antibody response in the context of autoimmunity. However, this approach can be applied to normal and malignant cell types and is amenable to scaling for high-throughput drug screening or application to commercially available compound libraries such as FDA approved compounds and kinase libraries. We believe this approach could be applied to identify separate functional and transcriptional units that can be targeted by drug combinations to develop powerful synergistic outcomes at the cellular level. We believe that the widespread application of this integrated approach can drive fundamental discoveries and uncover ways to modulate gene-expression networks through small molecule mediated targeting to control and manipulate cellular phenotypes.

## Methods

### Mice

Mice of 8-12 weeks of age were used for experiments for both in vitro and in vivo experiments. Constitutive cas9 mice were kindly provided by Marco Herold (WEHI) [37]. Conditional CD23^cre^ mice and Ezh2^fl/fl^ mice were kindly provided by Steve Nutt and Rhys Allan respectively (WEHI, Parkville, Victoria, Australia) [38, 39]. Blimp-1 GFP reporter mice were used were previously described [14]. Mice were maintained in specific pathogen-free conditions at the WEHI animal facilities (Parkville, Victoria), and experiments were performed in accordance with WEHI animal ethics committee regulations.

### Murine B cell isolation and culture

Purified naïve splenic B cells were isolated from mice by first using a percoll (GE Healthcare, IL, USA) gradient (80/65/50% in PBS, cells collected from 80/65% interface), followed by negative isolation with a B cell isolation kit (Miltenyi Biotec), as described previously [9, 12, 40]. Cells were labeled with division tracking dye, Cell Trace Violet (CTV). Purity of B cell population was verified as >95% B220^+^ CD19^+^ by flow cytometry. Labeled B cells were stimulated with lipopolysaccharide (LPS) derived from Escherichia coli 026:B6 (15 µg/mL; Sigma). For in vitro differentiation studies, B cells were stained using antibodies to CD138 (clone 281-2, BD Pharmingen). Triplicate plates were set up on the first day and left undisturbed – one plate was analyzed at each timepoint. All lymphocytes were incubated at 37 °C with 5% CO_2_ and humidity control.

### Human B cell isolation and culture

Healthy human peripheral blood samples were obtained from the Volunteer Blood Donor Registry (VBDR) at WEHI, Victoria, Australia. Informed consent was obtained from all individual participants prior to inclusion in the study. All procedures performed involving human participants were approved by and in accordance with the ethical standards of Human Research Ethics Committees at Melbourne Health and WEHI (Approved projects 2009.162, 10/02) and with the 1964 Helsinki declaration and its later amendments or comparable ethical standards.

Human naive B cells were isolated from cryopreserved PBMCs using the EasySep Naïve B cell Isolation Kit according to manufacturer’s instructions and as previously described [40]. Post-enrichment purity of naïve (CD20^+^CD27^-^) B cells was >98%. Purified naïve B cells were cultured in B-cell medium (RPMI 1640 supplemented with 10% fetal calf serum (FCS; Invitrogen Life Technologies), 10 mM HEPES, pH7.4 (Sigma-Aldrich), 0.1 mM nonessential amino acid solution (Sigma-Aldrich), 1 mM sodium pyruvate (Invitrogen Life Technologies), 60 mg/mL penicillin, 100 mg/mL streptomycin, 40 mg/mL transferrin (sigma-Aldrich) and 20 mg/mL Normocin (Invitrogen) and stimulated with 100 ng/mL megaCD40L (CD40L, Enzo) and IL-21 (50 ng/mL, Peprotech) in the presence of either 2 µM, 4 µM, 8 µM, or 16 µM of the EZH2 methyltransferase inhibitor GSK126 (Selleck Chem, TX, U.S.A.). GSK126 was added directly to the culture medium at 0 hr. Cells were cultured for 120 hr, collected, stained with a panel of monoclonal antibodies to assess differentiation and isotype switching (CD20 cat #563782, CD27 cat #8346807, CD38 cat #551400, IgA cat #130099220, IgG cat #563247 and IgM cat #564622) and the proportion of isotype switched and differentiated antibody secreting cells determined as previously described [29, 41]. Secreted IgM, IgG and IgA levels in culture supernatants were quantified by Ig Heavy chain specific ELISAs as previously described [29, 41].

### Quantitative analysis

Absolute cell number was determined with the addition of 1 × 10^4^ calibration beads directly to cells prior to analysis. 0.2 µM Propidium iodide (PI) was also added with the beads to identify dead cells by exclusion. Ratio of live cells to beads was measured by flow cytometry to determine the absolute live cell number in cell culture. Cohort number calculation was performed as described [11, 13].

### MAC-sequencing samples preparation and analysis

CTV labelled B cells were cultured with various compounds (1 µM final concentration) with LPS. At 24 hr or 72 hr, half of the samples were harvested for flow cytometry analysis to determine total cell number, survival, proliferation and ASC differentiation, measured by CD138 expression. 5 × 10^3^ cells from each well were aliquoted into a separate 96-well plate, washed in ice-cold PBS twice and centrifuged (1400 rpm at 4 °C for 4 min). Supernatant was removed, and cell pellets were frozen at −80 °C. Library preparation was performed by the addition of 15 µl lysis buffer into each well of a 96-well plate containing cell pellets and incubated at room temperature for 15 min under agitation (900 rpm). 12.5 µl of cell lysate was transferred into each well of a new 96-well plate previously prepared with 1 µl of 10 nM well-specific RT MAC-seq primer and 7.5 µl RT mix; the RT mix contains a TSO primer and external ERCC RNAs for normalization. The mixture was incubated for 2 hr at 42 °C to create well-barcoded full-length cDNA, and then all the wells of a plate were combined into a single tube. Concentration and clean-up were done with DNA Clean & ConcentratorTM-100 (Zymo Research), and RNAClean XP (Beckman Coulter) and each plate were eluted in 22 µl nuclease-free water. The purified cDNA was pre-amplified with KAPA HiFi HotStart ReadyMix (Roche), and MAC-seq PreAmp PCR primer and the quality checked on a D5000 Screentape (TapeStation, Agilent). One barcoded library was prepared per plate using TD buffer and TDE1 enzyme (Illumina) for tagmentation and KAPA HiFi HotStart Ready Mix (Roche) and custom primers (MAC-seq P5 PCR and MAC-seq Indexing Mix) for amplification. Libraries were purified with DNA AMPure XP (Beckman Coulter), quality checked on a DNA1000 tape (TapeStation, Agilent) and quantity verified by qPCR. Two indexed libraries were sequenced on a NextSeq 500 instrument (Illumina) using a custom sequencing primer (MAC-seq Read primer) and a High Output Kit v2.5 75 Cycles (Illumina) with paired-end configuration (25 base pairs for read 1 and 50 base pairs for read 2). Paired-end reads were demultiplexed using bcl2fastq (v2.17.1.14) and resulting FASTQ files were quality checked using fastqc (v0.11.6) and read 2 (R2) was trimmed 15 bp from the 5′ end to remove primer bias using cutadapt (v2.1; −u 15). R2 FASTQ files of paired-end reads were demultiplexed according to well barcodes (supplementary table 9) and filtered for PCR duplicates using Unique Molecular Identifiers (UMIs), both present in read 1 (R1) using the scruff 9 R (v4.0.2) package dumultiplex function (bcStart = 1, bcStop = 10, bcEdit = 0, umiStart = 11, umiStop = 20, keep = 35, minQual = 20, yieldReads = 1e + 06). R2 FASTQ files were then mapped to the GRCh37/hg19 genome and ERCC sequences using alignRsubread (unique = FALSE, nBestLocations = 1, format = “BAM”) and resulting BAM files were used to count unique R2 reads mapping to exonic genomic intervals and ERCC sequences using a combined hg19/ERCC GTF file with countUMI (umiEdit = 0, format = “BAM”, cellPerWell = 1. Both functions are from the scruff R package. Gene expression counts were normalized to library size. Subsequent count processing was performed using the Seurat R package (v3.2.1) 10, where lowly expressed genes were filtered, and counts were normalized for latent variables including plate, well row and column, using the SCTransform function. SCTransformed scaled gene RNA expression values were then used for PCA, where shared-nearest-neighbours (SNN) network was calculated using the top 10 Principal Components with the FindNeighbours function using default parameters. Drug-treatment clusters were subsequently identified with the Louvain algorithm using a resolution parameter of 2. Uniform Manifold Approximation and Projection (UMAP) values were also calculated using the top 10 Principal Components with the RunUMAP function using default parameters. Differential gene testing relative to treatment controls was performed using a hurdle model (MAST) and a logFC threshold of 0 with the FindMarkers Function. Area Under the Curve (AUC) scores for each drug treatment and gene lists indicated was calculated using all expressed genes with the R AUCell Package (v0.10.0). *ggplot2* (version 3.2.1) was used to visualize the data. Abundance of cell types was performed with Cibersortx [42] using bulk RNA-sequencing data published by Scharer et al. (GSE97696) [43].

### Enzyme-linked ImmunoSorbent Assay (ELISA)

Supernatant was removed from lymphocyte cultures and stored at −20 °C until ELISA analysis. 96-well ELISA plates (Sigma-Aldrich) were coated with corresponding plate coat antibody (Southern Biotech), diluted in PBS and incubated overnight at room temperature in humid conditions. Plates were then washed in PBS + Tween-20, PBS and distilled water. Supernatant samples and standards (Sigma) were titrated, diluted in block solution. Plates were incubated at room temperature in humid conditions overnight. Plates washed as before. Appropriate detection antibodies (Southern Biotech) were diluted in block solution and added to each well. Plates were incubated for 4 hr at room temperature in humid conditions. Plates were washed as before, and substrate solution was added into each well. Plates were left to develop for 30–45 min at room temperature in humid conditions, protected from light. Colour development was analyzed on VersaMax ELISA microplate reader (Molecular Devices), using wavelengths 415 minus 492.

### ELISpot and analysis

ELISpot was performed as previously described [44]. Briefly, nitrocellulose membranes of 96-well filtration plates (Millipore, Bedford, MA) were coated with NP_4_BSA or NP_20_BSA at 25 µg/mL in PBS for at least 4 hr. Plates were washed and cultured overnight with 1 × 10^5^ cells per well at 37 °C in 5% CO_2_. ELISpots were developed using HRPO goat anti-mouse IgG1 (as in ELISA) followed by filtered 3-amino-9-ethyl carbazole substrate (Sigma) at 250 µg/mL in 0.1 M acetate buffer, pH 5.0, 0.03% H_2_O_2_. The plates were washed in water, and the developed spots were analyzed using a custom ImageJ Macro script in run in FIJI, with MorpholibJ suite plugins [45]. Code is available on https://github.com/DrLachie/ElispotCounter. Briefly, all well images were opened as a stack and then registered to ensure all wells were centred. A median projection was performed on the stack to produce a clean background image devoid of spots, each well image was then compared to this background to enhance the contrast of the spots and allow simple thresholding for intensity and size to be performed. All detected spots were marked and saved as mask image for validation by the researchers, and number of spots and total area of detected spots was recorded.

### NP-immunization and analysis

NP-KLH immunization was performed as previously described [5, 20]. Briefly, 8–10 weeks old female C57BL/6 mice received a single intraperitoneal injection of 100 µg nitrophenyl coupled to keyhole limpet haemocyanin (NP-KLH) at a ratio of 21:1 and precipitated onto alum, prepared as described. Mice were treated with epigenetic modifying compounds five days post-immunization for seven days. Serum and spleen were harvested on day 14 for analysis. To determine immune response to NP immunization, single-cell suspensions were stained as described using antibodies to the following surface molecules: CD38 (clone:NIMR-5, in-house), CD19 (clone:1D3, cat #BD552854), IgM (clone:331.12, in-house), IgD (clone:11–26 C, in-house), Gr-1 (clone:RB6-8C5, in-house), CD138 (clone:281.2, cat #BD564068) and IgG1 (clone:X56, cat #BD550874). NP binding was detected as described [46].

### In vivo therapy

8–10 weeks old female C57BL/6 mice that were subjected to NP-immunization were treated with specified concentrations of GSK126, made up with 20% Captisol five days post NP-immunization. GSK126 was given daily for seven days via intraperitoneal injection. Organs were harvested 14 days post-immunization. Analysis was performed as above.

### Preparation of splenic sections

Splenic samples were performed as previously described [5]. Briefly, spleens are harvested and embedded in Tissue-Tek OCT compound (Miles) by flash-freezing in 2-methylbutane (Sigma-Aldrich) cooled with dry ice. The frozen tissues were stored at −80 °C until sectioning. Seven-micrometer sections were cut on a cryostat (Microm HM550) and mounted onto gelatin-coated slides. Sections were allowed to air-dry for 10 min, fixed in ice-cold acetone for 10 min, air-dried and stored at −20 °C until staining.

### Immunohistochemical staining of splenic sections and analysis

Spleen sections were rehydrated with PBS and endogenous peroxidase activity was blocked by 10 min incubation with 0.3% H_2_O_2_ before staining. The sections were then washed and blocked with 3% FCS + PBS for 30 min. Sections were stained with antibodies GL7 (GL7, in-house), IgD(1126 C, in-house), CD3(17A2, in-house) and B220(RA3-6B2, in-house). Images were acquired by upright Zeiss 880 microscope as configured as previously described [47]. Briefly, signal was detected using solid-state lasers (405, 561, 594 and 633 nm) and an argon laser (458, 488, 514 nm). Signal was visualized with W Plan-Apochromat ×20 DIC water immersion lens (1.0 N.A). An adjustable 32 channel spectral GaAsP detector was used to collect 405, 488, 594 and 647 signals. Zen Black 2012 software was used to stitch multiple images of whole spleen sections and images were quantified with ImageJ (NIH).

### RNA-sequencing and analysis

RNA sequencing was performed as previously described [19].3’mRNA-sequencing libraries were prepared from 100 ng of total RNA using the QuantSeq 3′ mRNA-Seq Library Prep Kit (Lexogen) according to the manufacturer’s instructions and sequenced on the NextSeq 500 (Illumina). The single-end 75 bp were demultiplexed using CASAVAv1.8.2 and Cutadapt (v1.9) was used for read trimming [48]. The trimmed reads were subsequently mapped to the mouse genome (mm10) using HISAT2 [49]. FeatureCounts from the Rsubread package (version 1.34.7) was used for read counting after which genes without a counts per million reads (CPM) in at least 3 samples were excluded from downstream analysis [50, 51]. Count data were normalized using the trimmed mean of M-values (TMM) method and differential gene expression analysis was performed using the limma-voom pipeline (limma version 3.40.6) [50, 52, 53]. Comparisons between different doses (4 µM vs 0 µM, 8 µM vs 0 µM and 8 µM vs 4 µM) for different timepoints (6 hr, 71 hr, 95 hr) and comparisons between different timepoints (6 hr vs 0 hr, 71 hr vs 0 hr and 95 hr vs 0 hr) were made for 0 µM dose. Adjustment for multiple testing was performed per comparison using the false discovery rate (FDR) method [54]. Heatmaps of logCPM were generated using *pheatmap*. GSEA-2.2.2 was used for Gene set enrichment analysis (GSEA) [55, 56]. The R/Bioconductor package *TCseq* (version 1.8.0) was used to perform time course clustering analysis on normalized log-transformed CPM using k-means clustering and *ggplot2* (version 3.2.1) was used to plot the cluster-specific trends [57, 58].

### ChIP-sequencing and analysis

ChIP-sequencing was performed as previously described [59]. 2 µg anti-H3K27me3 antibody (cat# ab6002) was used for immunoprecipitation. DNA product was purified using Zymo ChIP DNA Clean and Concentrator Kit. ChIP-enriched DNA was processed using TruSeq Sample Prep Kit (Illumina) according to manufacturer’s instructions and sequenced on HiSeq2500 (Illumina). 20 million single-end 50 bp reads were generated per sample. CASAVA (v1.8.2) was used for demultiplexing. The Fastq files generated were aligned to the mouse reference genome (mm10) using bowtie (v2.2.3) [60]. Samtools (v1.3) was used for manipulation of SAM and BAM files and MACS (V2.0.10) was used for peak calling [61, 62]. HOMER (v4.8.3) was used for quantification and annotation of the ChIP-Seq datasets and data were visualized using R, IGV and deeptools (v2.5.3) [63]. *FeatureCounts* from the R *Rsubread* package (version 1.34.7) was used to quantify gene body and promoter counts. Regions with low counts were filtered out using *filterByExpr* function in *edgeR* (version 3.26.8) [64]. Count data was normalized using trimmed mean of M-values (TMM) method and differential binding analysis was performed using the *limma-voom* pipeline [52, 53]. Comparisons between different doses (4 μM vs 0 μM, 8 μM vs 0 μM and 8 μM vs 4 μM) for different time points (48 hr and 72 hr) and comparisons between different time points (48 hr vs 0 hr and 72 hr vs 0 hr) for 0 μM were made. Adjustment for multiple testing was performed per comparison using the FDR method [54].

### Analysis of public data

For RNA-sequencing data published by Shi et al. (GSE60927), count table was downloaded prior to analysis. Genes with a count per million (CPM) in at least three samples were included downstream analysis [50, 51]. Count data were normalized using the trimmed mean of M-values (TMM) method, and differential gene expression analysis was performed using the limma-voom pipeline (*limma* version 3.40.6) [50, 52, 53]. Data were visualized using Prism8. Fastq files for ChIP-sequencing data in Figure 6 were obtained from GSE71698. The Fastq files generated were aligned to the mouse reference genome (mm10) using bowtie (v2.2.3) [60]. Samtools (v1.3) was used for manipulation of SAM and BAM files, and MACS (V2.0.10) was used for peak calling. Data were visualized using IGV.

### CRISPR gRNA library

gRNAs were obtained from the Sanger Arrayed Whole Genome Lentiviral CRISPR Library (Sigma-Aldrich). This library consists of gRNAs within the third generation lentiviral plasmid vector U6-gRNA:PGK-PuroR-2A-tagBFP.

### Production of lentiviral vectors

Lentiviral production was performed using Fugene6 (Promega). 293T cells were plated at 2 × 10^4^ cells per well of a 96-well plate and incubated overnight before the addition of the transfection mixture. The transfection mixture consisted of 0.6 µg pMDL, 0.4 µg pRSV-Rev and 0.6 µg gRNA plasmid, in combination with either 0.4 µg Eco or 0.4 µg pCMV-VSV-G. Fugene6 was added to the transfection mixture at a ratio of 6:1 to the total DNA content and incubated for 15 min at room temperature before addition to 293T media. Following the addition of the transfection mixture, 293Ts were incubated for a further 48 hr, at which point the lentivirus containing supernatant was frozen and stored at −80 °C until use.

### Lentiviral transduction of primary mouse B cells

Non-tissue culture treated 96-well plates were coated for 16 hr with retronectin at 32 µg/mL and blocked with PBS + 2% BSA prior to the addition of cells and lentivirus supernatant. Plates were centrifuged at 1200 rpm at 28 °C for 90 min. Following lentiviral transduction, viral supernatant was removed, and cells were cultured in LPS derived from Escherichia coli 026:B6 (15 µg/mL; Sigma) and IL-4 (500 U/mL; WEHI) for 48 hr prior to GSK126 treatment. Expression of CD138 was measured via flow cytometry two days post GSK126 treatment.

### CRISPR screen analysis

CRISPR screen output is measured using flow cytometry. For the analysis to determine genes essential for differentiation in the untreated samples (Fig. 4h), the changes in CD138 expression between uninfected and guide infected samples were quantified and ranked based on the difference in CD138. To determine the targets of Ezh2 inhibitor in Fig. 4i, the difference in CD138 expression of the cells infected with guides between untreated and GSK126 were quantified and ranked based on the difference in CD138. A cut-off score of -1 z-score is used to determine gene targets of Ezh2 mediated differentiation. Data were visualized using R.

### Statistics

The sample size required for the experiments was estimated based on the results of preliminary data. Analysis on germinal centre was performed blinded, where the genotype of the mice were not revealed to the researcher performing the analysis. Randomisation for animal experiments were not necessary due to the nature of the experiments. Statistical differences between the means of two data groups was determined by using two-tailed unpaired Student’s *t* test, and *p* values < 0.05 were considered significant. Multiple group comparisons were performed using ANOVA with a Bonferroni correction, *p* values < 0.05 were considered significant.

## Supplementary information

Figure S1

Figure S2

Figure S3

Figure S4

Supplementary Table 1

Supplementary Table 2

Supplementary Table 3

Supplementary figure legends

ptr-authorship form

Reproducibility Checklist

## Data Availability

The datasets generated during this study are available at GEO: GSE185326.
