# Supplementary figures and images for: Epigenetic modulators of B cell fate identified through coupled phenotype-transcriptome analysis
Source: Cell Death Differ. 2022 Jul 13;29(12):2519–30. doi: 10.1038/s41418-022-01037-5 (PMC9751284; doi:10.1038/s41418-022-01037-5)

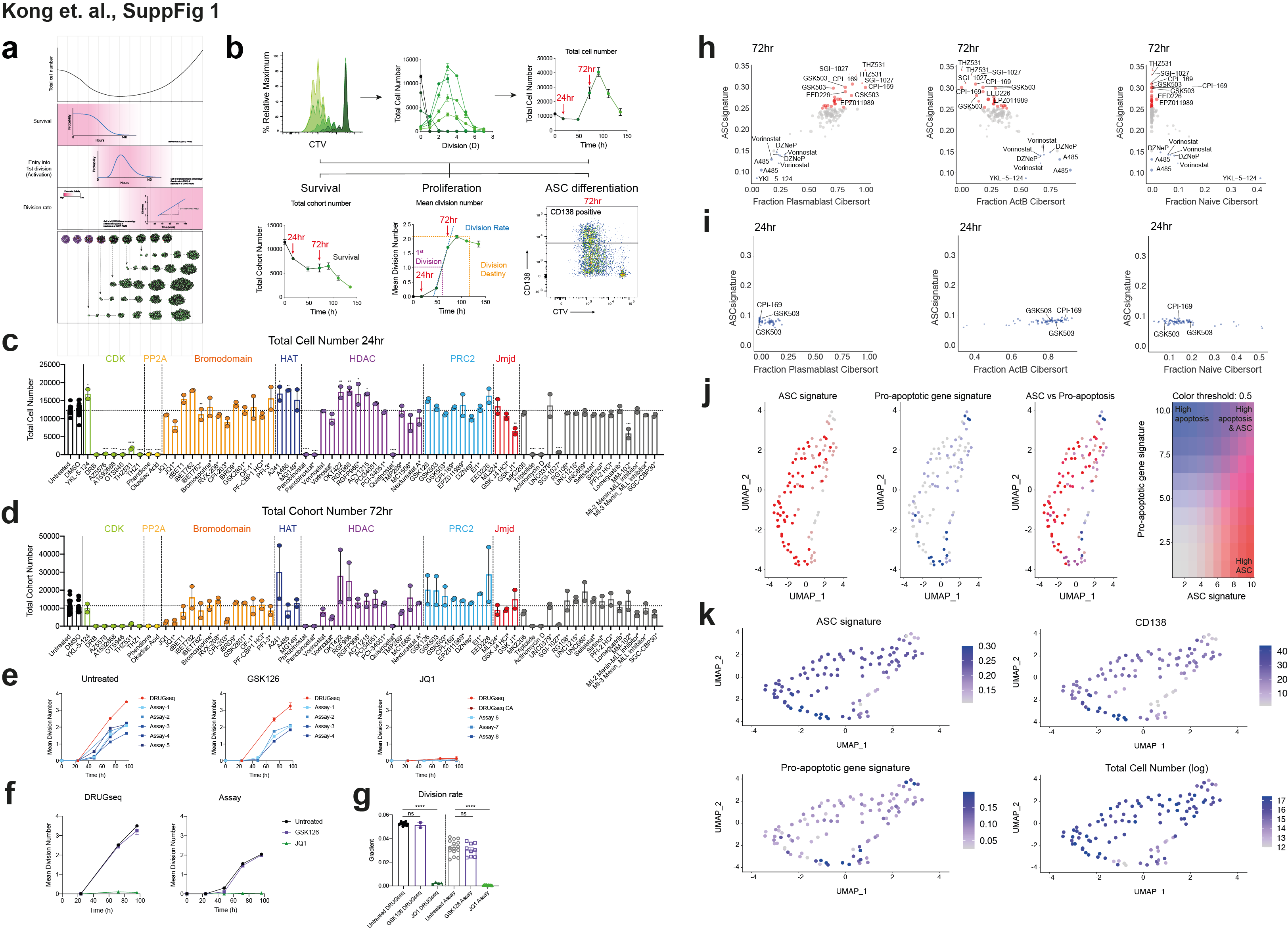

Supplement: Supplementary file 1 — Figure S1 [file 41418_2022_1037_MOESM1_ESM.png]

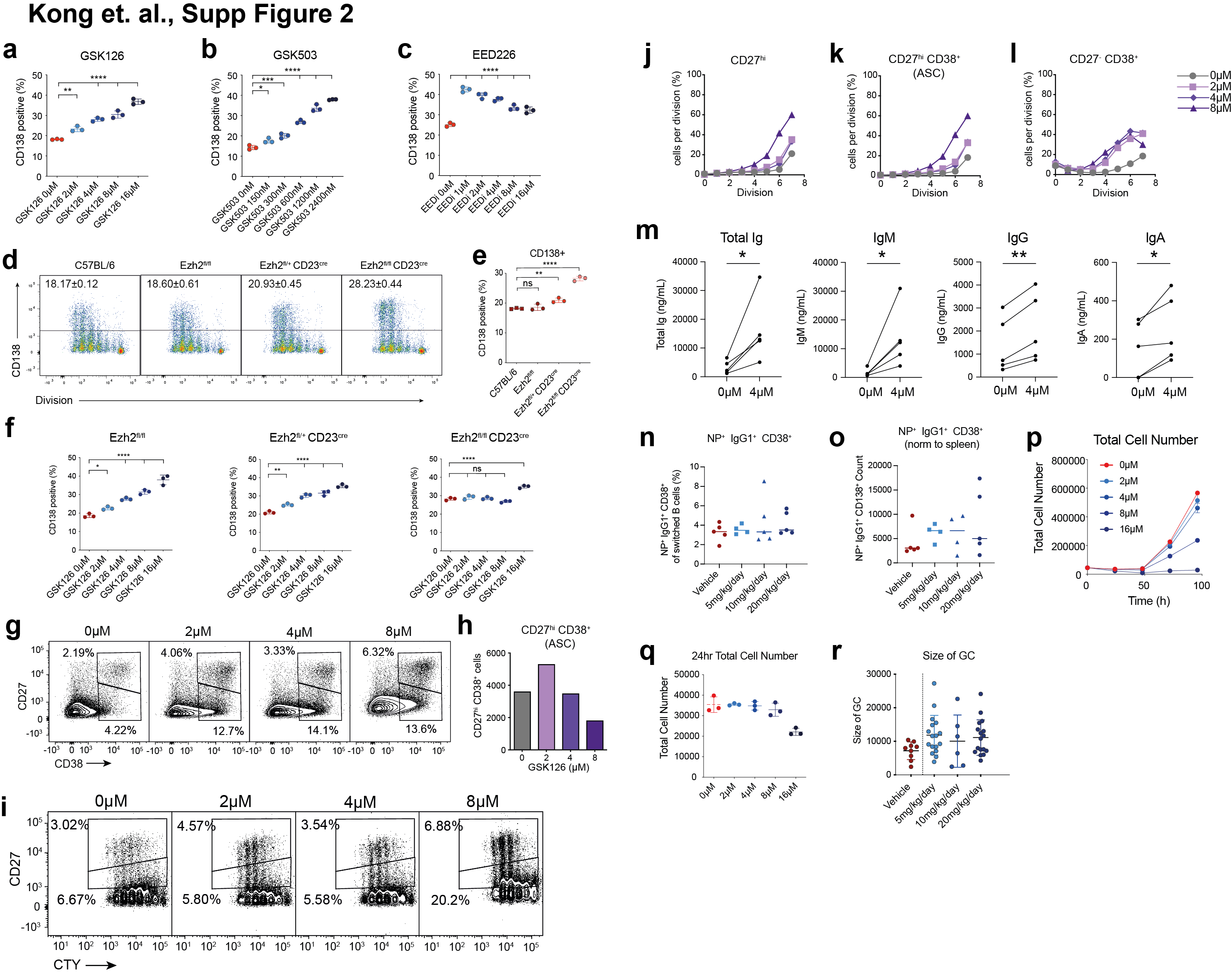

Supplement: Supplementary file 2 — Figure S2 [file 41418_2022_1037_MOESM2_ESM.png]

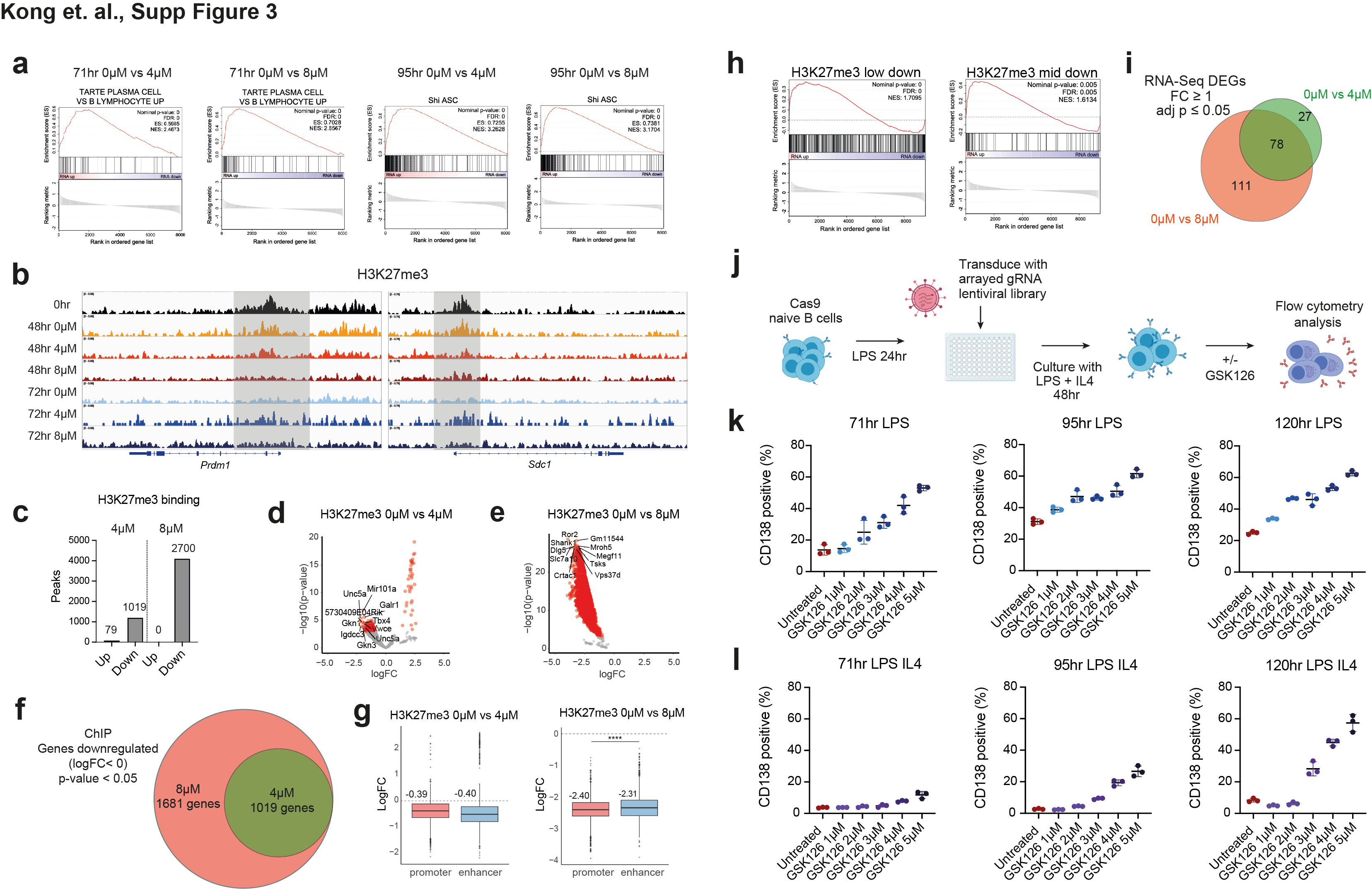

Supplement: Supplementary file 3 — Figure S3 [file 41418_2022_1037_MOESM3_ESM.png]

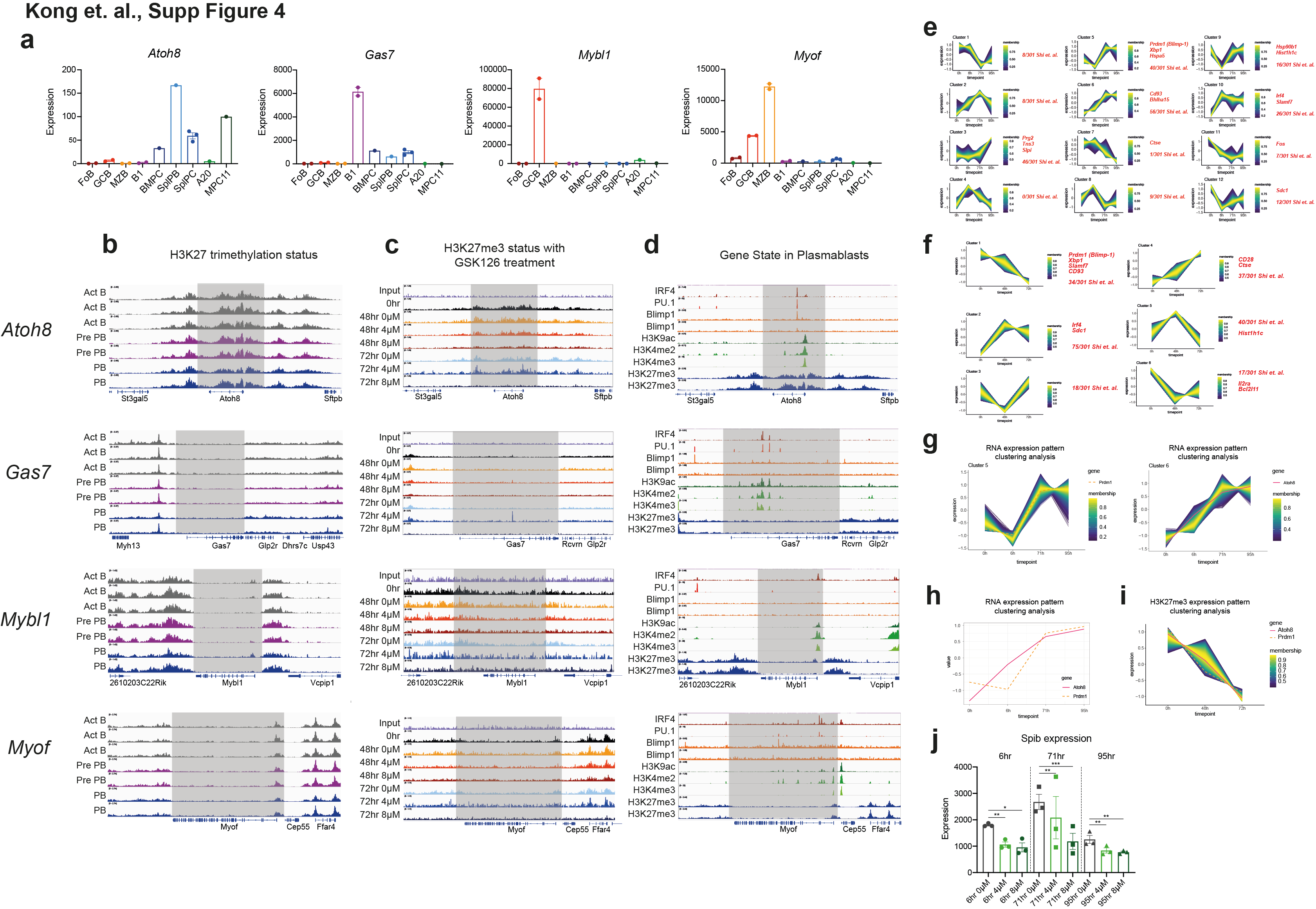

Supplement: Supplementary file 4 — Figure S4 [file 41418_2022_1037_MOESM4_ESM.png]
