# Supplementary material for: Epigenetic modulators of B cell fate identified through coupled phenotype-transcriptome analysis
Source: Cell Death Differ. 2022 Jul 13;29(12):2519–30. doi: 10.1038/s41418-022-01037-5 (PMC9751284; doi:10.1038/s41418-022-01037-5)
Supplement: Supplementary file 8 — Supplementary figure legends [file 41418_2022_1037_MOESM8_ESM.docx]

**Supplementary Figure Legends**

**Figure S1 MAC-seq to identify the effects and target genes of EMCs.**

The Cyton Model is a quantitative theoretical framework built from division tracking datasets that separate lymphocyte behaviour into individual measurable components, resulting in the typical response curve shown in (**a**). In this model, three key parameters are used to describe the overall immune response *in vitro* following stimulation. These parameters have been tested at the population and single-cell level using flow cytometry and single-cell time-lapse microscopy studies. Through this analysis, the lymphocyte response has been reduced to the following parameters: survival of the cells, time taken for cells to enter division and division rate of cells. The combined effect of these parameters on the founding cell population and cells recruited into division is represented in the schematic in the bottom panel. (**b**) Schematic for cohort calculation to examine total cell number, total cohort number (survival), mean division number (proliferation) and ASC differentiation. CTV-labeled murine naïve B cells were cultured in LPS with the addition of various compounds. (**c**) Total cell number at 24hr post culture. (**d**) Total cohort number at 72hr post culture. (**e**) Mean division number for untreated, GSK126 and JQ1 samples measured using MAC-seq with reduced timepoints and assays with multiple timepoints. (**f**) Mean division number for MAC-seq assay and multiple timepoint assays. (**g**) Division rate calculated for MAC-seq and assays. (**h**) Correlation between ASC signature genes and the fraction of plasmablasts, activated B cells, and naïve B cells at (**h**) 72hr and (**i**) 24hr determined using Cibersortx. (**j**) UMAP plots highlighting ASC gene expression, pro-apoptotic gene expression and ASC signature gene expression and pro-apoptotic signature gene expression overlapped. (**k**) UMAP plot highlighting the expression of ASC signature, CD138 expression, pro-apoptotic gene signature and total cell number (log). Significant differences in **c-e** were determined using ANOVA with Bonferroni corrections. * p≤0.05, ** p≤0.01, *** p≤0.001, **** p≤0.0001.

**Figure S2 PRC2 inhibition increases ASC differentiation.**

CTV-labeled murine B cells were stimulated with LPS. Percentage of CD138 positive cells three days post-stimulation with (**a**) GSK126, (**b**) GSK503, and (**c**) EED226. (**d**) CD138 expression three days post activation for B cells isolated from C57BL/6, Ezh2^fl/fl^, Ezh2^fl/+^ CD23^cre^ and Ezh2^fl/fl^ CD23^cre^ and proportion of CD138 positive cells were quantified in (**e**). (**f**) Proportion of CD138 positive B cells from Ezh2^fl/fl^, Ezh2^fl/+^ CD23^cre^ and Ezh2^fl/fl^ CD23^cre^ three days post-stimulation with indicated concentrations of GSK126. CTY-labeled human naïve B cells were cultured with CD40L (100 ng/mL) +IL-21 (50 ng/mL) for 120hr, alone or in the presence of GSK126 at either 2, 4, 8, or 16µM as indicated. (**g**) Flow cytometry plots of CD27 vs CD38. (**h**) Total CD27^hi^ CD38^+^ ASC cells in (**g**) were quantified. (**i**) Flow cytometry plots of acquisition of CD27 expression vs CTY. Percentage of (**j**) CD27^hi^ cells, (**k**) CD27^hi^ CD38^+^ cells and (**l**) CD27^-^ CD38^+^ cells per division. (**m**) Amount of immunoglobulin (total, IgA, IgG and IgM) detected in cultures after 120hr (ng/mL) as measured by Ig-specific ELISA assays as indicated. C57BL/6 mice were immunized with NPKLH-Alum and treated with doses of GSK126 for seven days starting on the fifth day post-immunization. Spleen and serum samples were analyzed 14 days after immunization. (**n**) Proportion and (**o**) total cell number normalized to the spleen for antigen-specific memory cells in the spleen were quantified. (**p**) Total number of murine B cells upon LPS stimulation and treatment with various concentrations of GSK126. (**q**) Total number of murine B cells at 24hrs post stimulation with LPS and indicated concentration of GSK126. (**r**) The size of GC of mice immunised with NPKLH-Alum and treated with indicated doses of GSK126 was quantified as defined in materials and methods. Vehicle, n=5, 5mg/kg/day GSK126 treated group, n=4, 10mg/kg/day and 20mg/kg/day GSK126 treated groups, n=5. Data in **e** are representative plots from triplicate samples. Error bars in **a-c**, **e-f, h** and **j-q** denote mean ± s.e.m. All data are representative of three independent experiments. All statistical tests were performed in comparison to the untreated control. Data shown in **g-l** are from one healthy donor, representative of two unrelated healthy donors. Data shown in **m** are from five unrelated healthy donors. Significance differences in **a-c** and **e-f** were determined using ANOVA Bonferroni corrections. Significant differences in m were determined using paired student T test. * p≤0.05, ** p≤0.01, *** p≤0.001, **** p≤0.0001.

**Figure S3 RNA-seq and ChIP-seq to validate MAC-seq data for Ezh2 inhibitor GSK126.**

3’ RNA-Seq was performed on LPS stimulated murine B cells at various timepoints with indicated concentrations of GSK126. (**a**) GSEA of genes differentially regulated by LPS activated B cells between untreated, and GSK126 treated B cells (4µM or 8µM) at 71hr and 95hr. Antibody secreting cell gene signature was obtained from Shi *et al*. Nature Immunology (2015) (GSE60927) and Tarte Plasma cell vs B lymphocyte gene signature (MSigDB: M4552). ChIP-sequencing was performed to examine the H3K27me3 occupancy upon treatment with GSK126. (**b**) H3K27me3 peaks of 0hr, 48hr (0µM, 4µM and 8µM GSK126) and 72hr (0µM, 4µM and 8µM GSK126) at *Prdm1* and *Sdc1* regions. Grey boxes indicate promoter regions bound by H3K27me3. (**c**) Upregulated and downregulated H3K27me3 peaks with 4µM and 8µM GSK126 at 72hr. Volcano plot for differentially bound H3K27me3 peaks for (**d**) 0µM vs 4µM and (**e**) 0µM vs 8µM at 72hr. (**f**) Venn diagram showing the genes with H3K27me3 downregulation for 0µM vs 4µM and 0µM vs 8µM at 72hr with cut off p-val≤0.05. Fold change of peaks for (**f**) 0µM vs 4µM and (**g**) 0µM vs 8µM for the promoter and enhancer regions. (**h**) GSEA analysis of genes in low down group (genes with H3K27me3 logFC between -1 and 0) and mid down group (genes with H3K27me3 logFC between -2 and -1) in relation to gene expression upon GSK126 treatment. (**i**) Venn diagram for DEGs that are upregulated for 0µM vs 4µM GSK126 and 0µM vs 8µM GSK126. (Cut-off values: FC≥ 1 and adj p-val ≤0.05) (**j**) Schematic showing the workflow for CRISPR-screen. Proportion of CD138 positive cell of (**k**) LPS (15µg/mL) and (**l**) LPS (15µg/mL) + IL-4 (100U/mL) activated B cells at 71hr, 95hr and 120hr with indicated concentrations of GSK126. Data in **k** and **l** are mean ± s.e.m of triplicate samples and are representative of three independent experiments.

**Figure S4 Expression and H3K27me3 pattern of *Atoh8*, *Gas7*, *Mybl1* and *Myof*.**

(**a**) Expression of *Atoh8*, *Gas7*, *Mybl1* and *Myof* in different subsets of B cells (Follicular B cells, GC B cells, marginal zone B cells, B1 cells, bone marrow plasma cells, splenic plasmablasts and splenic plasma cells), A20 (GC cell line) and MPC11 (plasmacytoma cell line). (**b**) H3K27me3 at *Atoh8, Gas7*, *Mybl1* and *Myof* in activated B cells (Act B), pre-plasmablasts (Pre PB) and plasmablasts (PB). (**c**) H3K27me3 at 0hr, 48hr and 72hr upon treatment with GSK126 at indicated concentrations at *Atoh8*, *Gas7*, *Mybl1* and *Myof.* (**d**) Binding of IRF4, PU.1, Blimp-1, H3K9ac, H3K4me2 and H3K27me3 in plasmablasts at *Atoh8, Gas7*, *Mybl1* and *Myof* regions. (**e**) Clustering analysis of RNA expression pattern in untreated samples at 0hr, 6hr, 71hr and 95hr. (**f**) Clustering analysis of H3K27me3 expression pattern in untreated samples at 0hr, 48hr and 72hr. Labels in **e-f** indicate the number of genes in each cluster that are in ASC gene signature by Shi *et al*. (**g**) Clustering analysis of RNA expression pattern at 0hr, 6hr, 71hr and 95hr highlighting *Prdm1* (Cluster 5) and *Atoh8* (Cluster 6). (**h**) Temporal expression pattern of *Prdm1* and *Atoh8* overlapped. (**i**) Clustering analysis of H3K27me3 expression pattern at 0hr, 6hr, 71hr and 95hr (Cluster 1) highlighting *Prdm1* and *Atoh8*. (**j**) **Expression of** *Spib*. Data **a** were obtained from Shi et al. Nature Immunology (2015) (GSE60927). Data in **c**-**d** were obtained from GSE71698. Significance differences were determined using *limma*. * p≤0.05, ** p≤0.01, *** p≤0.001.

**Supplementary Table 1 Epigenetic modifying compounds used for MAC-seq.**

**Supplementary Table 2 Genes in RNA-seq and H3K27me3 ChIP-seq cluster analysis.**

**Supplementary Table 3 ASC (Shi *et al*) and pro-apoptotic gene signature.**
