# Supplementary material for: Epigenetic modulators of B cell fate identified through coupled phenotype-transcriptome analysis
Source: Cell Death Differ. 2022 Jul 13;29(12):2519–30. doi: 10.1038/s41418-022-01037-5 (PMC9751284; doi:10.1038/s41418-022-01037-5)
Supplement: Supplementary file 9 — ptr-authorship form [file 41418_2022_1037_MOESM9_ESM.pdf]

SPRINGER NATURE

Change of authorship request form - Journals (pre-acceptance)

Important information. Please read.

- This form should be used by authors to request any change in authorship (adding/deleting authors) including changes in corresponding authors. This form should not be used for name changes. Please fully complete all sections. Use black ink and block capitals and provide each author's full name with the given name first followed by the family name.

- By signing this declaration, all authors guarantee that the order of the authors are in accordance with their scientific contribution, if applicable as different conventions apply per discipline, and that only authors have been added who made a meaningful contribution to the work.

- Please note, in author collaborations where there is formal agreement for representing the collaboration, it is sufficient for the representative or legal guarantor (usually the corresponding author) to complete and sign the Authorship Change Form on behalf of all authors, **next to the added/removed author(s)**. **(Complete Section 3, followed by Section 6.)**
  In author collaborations where there is no formal agreement for representing the collaboration and **there are more than 10 authors**, one may sign for all, provided the signer appends correspondence that attests that each of the authors have agreed to the change **and the added/removed authors sign the form**. **(Complete Section 3, followed by Section 6.)**

- Please note, we cannot investigate or mediate any authorship disputes. If you are unable to obtain agreement from all authors (including those who you wish to be removed) you must refer the matter to your institution(s) for investigation. Please inform us if you need to do this.

- If you are not able to return a fully completed form within **30 days** of the date that it was sent to the author requesting the change, we may have to withdraw your manuscript. We cannot publish manuscripts where authorship has not been agreed by all authors (including those who have been removed).

- Incomplete forms will be rejected.

- Please return/upload this form, fully completed, to the Journals Editorial Office. The Journal and/or Publisher will consider the information you have provided to decide whether to approve the proposed change in authorship. We may decide to contact your institution for more information or undertake a further investigation, if appropriate, before making a final decision.

SPRINGER NATURE Springer Nature is one of the world's leading global research, educational and professional publishers, created in May 2015 through the combination of Nature Publishing Group, Palgrave Macmillan, Macmillan Education and Springer Science+Business Media.

SPRINGER NATURE

Change of authorship request form - Journals (pre-acceptance)

Section 1: Please provide the current title of manuscript

Manuscript ID no.: CDD-21-2888RR

Title: Epigenetic modulators of B cell fate identified through coupled phenotype-transcriptome analysis

Section 2: Please provide the previous authorship, in the order shown on the manuscript before the changes were introduced. Please indicate the corresponding author by adding (CA) behind the name.

	First name(s)	Family name	ORCID or SCOPUS id, if available
1st author	Isabella	Kong	
2nd author	Stephanie	Trezise	
3rd author	Amanda	Light	
4th author	Izabela	Todorovski	
5th author	Gisela	Mir Arnau	
6th author	Sreeja	Gadipally	
7th author	David	Yoannidis	
8th author	Kaylene	Simpson	
9th author	Xueyi	Dong	
10th author	Lachlan	Whitehead	

Please use an additional sheet if there are more than 10 authors.

SPRINGER NATURE Springer Nature is one of the world's leading global research, educational and professional publishers, created in May 2015 through the combination of Nature Publishing Group, Palgrave Macmillan, Macmillan Education and Springer Science+Business Media.

SPRINGER NATURE

Change of authorship request form - Journals (pre-acceptance)

Section 1: Please provide the current title of manuscript

Manuscript ID no.: CDD-21-2888RR

Title: Epigenetic modulators of B cell fate identified through coupled phenotype-transcriptome analysis

Section 2: Please provide the previous authorship, in the order shown on the manuscript before the changes were introduced. Please indicate the corresponding author by adding (CA) behind the name.

	First name(s)	Family name	ORCID or SCOPUS id, if available
11th author	Jessica	Tempany	
12th author	Amania	Sheikh	
13th author	Joanna	Groom	
14th author	Kelly	Rogers	
15th author	Marco	Herold	
16th author	Vanessa	Bryant	
17th author	Matthew	Ritchie	
18th author	Simon	Willis	
19th author	Ricky	Johnstone	
20th author	Philip	Hodgkin	

Please use an additional sheet if there are more than 10 authors.

SPRINGER NATURE Springer Nature is one of the world's leading global research, educational and professional publishers, created in May 2015 through the combination of Nature Publishing Group, Palgrave Macmillan, Macmillan Education and Springer Science+Business Media.

SPRINGER NATURE

Change of authorship request form - Journals (pre-acceptance)

Section 1: Please provide the current title of manuscript

Manuscript ID no.:	CDD-21-2888RR

Title:	Epigenetic modulators of B cell fate identified through coupled phenotype-transcriptome analysis

Section 2: Please provide the previous authorship, in the order shown on the manuscript before the changes were introduced. Please indicate the corresponding author by adding (CA) behind the name.

	First name(s)	Family name	ORCID or SCOPUS id, if available
21st author	Stephen	Nutt	
22nd author	Stephin	Vervoort (CA)	0000-0001-7459-126X
23rd author	Edwin	Hawkins (CA)	0000-0002-3686-8261
24th author			
25th author			
26th author			
27th author			
28th author			
29th author			
30th author			

Please use an additional sheet if there are more than 10 authors.

SPRINGER NATURE Springer Nature is one of the world's leading global research, educational and professional publishers, created in May 2015 through the combination of Nature Publishing Group, Palgrave Macmillan, Macmillan Education and Springer Science+Business Media.

Change of authorship request form - Journals (pre-acceptance)

Section 3: Please provide a justification for change. Please use this section to explain your reasons for changing the authorship of your manuscript, e.g. what necessitated the change in authorship? Please refer to the (journal) policy pages for more information about authorship. Please explain why omitted authors were not originally included and/or why authors were removed on the submitted manuscript.

Anthony Franchione performed additional experiments requested by the reviewers.

Section 4: Proposed new authorship. Please provide your new authorship list in the order you would like it to appear on the manuscript. Please indicate the corresponding author by adding (CA) behind the name. <u>If the Corresponding Author has changed, please indicate the reason under section 3</u>.

	First name(s)	Family name (this name will appear in full on the final publication and will be searchable in various abstract and indexing databases)	Affiliated institute	E-mail address
1st author	Isabella	Kong	The Walter and Eliza Hall Institute of Medical Research	kong.i@wehi.edu.au
2nd author	Stephanie	Trezise	The Walter and Eliza Hall Institute of Medical Research	strezise@mgh.harvard.edu
3rd author	Amanda	Light	The Walter and Eliza Hall Institute of Medical Research	light@wehi.edu.au
4th author	Izabela	Todorovski	Peter MacCallum Cancer Centre	izabela.todorovski@petermac.org
5th author	Gisela	Mir Arnau	Peter MacCallum Cancer Centre	gisela.mirarnau@petermac.org
6th author	Sreeja	Gadipally	Peter MacCallum Cancer Centre	sreeja.Gadipally@onjcri.org.au
7th author	David	Yoannidis	Peter MacCallum Cancer Centre	david.yoannidis@petermac.org
8th author	Kaylene	Simpson	Peter MacCallum Cancer Centre	kaylene.simpson@petermac.org
9th author	Xueyi	Dong	The Walter and Eliza Hall Institute of Medical Research	dong.x@wehi.edu.au
10th author	Lachlan	Whitehead	The Walter and Eliza Hall Institute of Medical Research	whitehead@wehi.edu.au

Please use an additional sheet if there are more than 10 authors.

SPRINGER NATURE Springer Nature is one of the world's leading global research, educational and professional publishers, created in May 2015 through the combination of Nature Publishing Group, Palgrave Macmillan, Macmillan Education and Springer Science+Business Media.

SPRINGER NATURE

Change of authorship request form - Journals (pre-acceptance)

Section 3: Please provide a justification for change. Please use this section to explain your reasons for changing the authorship of your manuscript, e.g. what necessitated the change in authorship? Please refer to the (journal) policy pages for more information about authorship. Please explain why omitted authors were not originally included and/or why authors were removed on the submitted manuscript.

Anthony Franchione performed additional experiments requested by the reviewers.

Section 4: Proposed new authorship. Please provide your new authorship list in the order you would like it to appear on the manuscript. Please indicate the corresponding author by adding (CA) behind the name. If the Corresponding Author has changed, please indicate the reason under section 3.

	First name(s)	Family name (this name will appear in full on the final publication and will be searchable in various abstract and indexing databases)	Affiliated institute	E-mail address
11th author	Jessica	Tempany	The Walter and Eliza Hall Institute of Medical Research	jessicatempany@gmail.com
12th author	Anthony	Franchione	The Walter and Eliza Hall Institute of Medical Research	francione.a@wehi.edu.au
13th author	Amania	Sheikh	The Walter and Eliza Hall Institute of Medical Research	sheikh.a@wehi.edu.au
14th author	Joanna	Groom	The Walter and Eliza Hall Institute of Medical Research	groom@wehi.edu.au
15th author	Kelly	Rogers	The Walter and Eliza Hall Institute of Medical Research	rogers@wehi.edu.au
16th author	Marco	Herold	The Walter and Eliza Hall Institute of Medical Research	herold@wehi.edu.au
17th author	Vanessa	Bryant	The Walter and Eliza Hall Institute of Medical Research	bryant.v@wehi.edu.au
18th author	Matthew	Ritchie	The Walter and Eliza Hall Institute of Medical Research	mritchie@wehi.edu.au
19th author	Simon	Willis	The Walter and Eliza Hall Institute of Medical Research	willis@wehi.edu.au
20th author	Ricky	Johnstone	Peter MacCallum Cancer Centre	ricky.johnstone@petermac.org

Please use an additional sheet if there are more than 10 authors.

SPRINGER NATURE Springer Nature is one of the world's leading global research, educational and professional publishers, created in May 2015 through the combination of Nature Publishing Group, Palgrave Macmillan, Macmillan Education and Springer Science+Business Media.

SPRINGER NATURE

Change of authorship request form - Journals (pre-acceptance)

Section 3: Please provide a justification for change. Please use this section to explain your reasons for changing the authorship of your manuscript, e.g. what necessitated the change in authorship? Please refer to the (journal) policy pages for more information about authorship. Please explain why omitted authors were not originally included and/or why authors were removed on the submitted manuscript.

Anthony Franchione performed additional experiments requested by the reviewers.

Section 4: Proposed new authorship. Please provide your new authorship list in the order you would like it to appear on the manuscript. Please indicate the corresponding author by adding (CA) behind the name. <u>If the Corresponding Author has changed, please indicate the reason under section 3</u>.

	First name(s)	Family name (this name will appear in full on the final publication and will be searchable in various abstract and indexing databases)	Affiliated institute	E-mail address
21st author	Philip	Hodgkin	The Walter and Eliza Hall Institute of Medical Research	hodgkin@wehi.edu.au
22nd author	Stephen	Nutt	The Walter and Eliza Hall Institute of Medical Research	nutt@wehi.edu.au
23rd author	Stephin	Vervoort (CA)	The Walter and Eliza Hall Institute of Medical Research	vervoort.s@wehi.edu.au
24th author	Edwin	Hawkins (CA)	The Walter and Eliza Hall Institute of Medical Research	hawkins.e@wehi.edu.au
25th author				
26th author				
27th author				
28th author				
29th author				
30th author				

Please use an additional sheet if there are more than 10 authors.

SPRINGER NATURE Springer Nature is one of the world's leading global research, educational and professional publishers, created in May 2015 through the combination of Nature Publishing Group, Palgrave Macmillan, Macmillan Education and Springer Science+Business Media.

SPRINGER NATURE Change of authorship request form - Journals (pre-acceptance)

Section 5: Author contribution, Acknowledgement and Disclosures. Please use this section to provide a new disclosure statement and, if appropriate, acknowledge any contributors who have been removed as authors and ensure you state what contribution any new authors made (if applicable per the journal or book (series) policy). Please ensure these are updated in your manuscript - after approval of the change(s) - as our production department will not transfer the information in this form to your manuscript.

New acknowledgements:

Not applicable

New Disclosures (financial and non-financial interests, funding):

No applicable

New Author Contributions statement (if applicable per the journal policy):

AJF performed human ELISA experiments.

State 'Not applicable' if there are no new authors.

SPRINGER NATURE Springer Nature is one of the world's leading global research, educational and professional publishers, created in May 2015 through the combination of Nature Publishing Group, Palgrave Macmillan, Macmillan Education and Springer Science+Business Media.

SPRINGER NATURE

Change of authorship request form - Journals (pre-acceptance)

	First name	Family name		Signature	Date
Corresponding author	Edwin	Hawkins (CA)	I agree to the proposed new authorship shown in section 4	[signature]	17/06/22
Corresponding author	Stephin	Vervoort (CA)	I agree to the proposed new authorship shown in section 4	[signature]	17/06/22

Please use an additional sheet if there are more than 10 authors.

In case of author collaborations with formal agreement:

	Name of consortium/consortia	First name	Family name		Signature	Date
Representative/legal guarantor				I agree to the proposed new authorship shown in section 4 /**and the addition/removal*of my name to the authorship list** /and the proposed change in corresponding author		

Both added/removed authors should complete the information in the first table under Section 6.

---- End of form ----

SPRINGER NATURE Springer Nature is one of the world's leading global research, educational and professional publishers, created in May 2015 through the combination of Nature Publishing Group, Palgrave Macmillan, Macmillan Education and Springer Science+Business Media.
